# Supplementary material for: Quantifying exchange forces of a spin spiral on the atomic scale
Source: Nat Commun. 2020 Mar 5;11:1197. doi: 10.1038/s41467-020-15024-2 (PMC7057993; doi:10.1038/s41467-020-15024-2)
Supplement: Supplementary file 1 — Supplementary Information [file 41467_2020_15024_MOESM1_ESM.pdf]

Supplementary Information

Quantifying exchange forces of a spin spiral on the atomic scale

Hauptmann et al.

Supplementary Note 1: Sample preparation and magnetic characterization of the tip

Supplementary Fig. 1 shows an overview image of the Mn monolayer on W(110) acquired with a mainly out-of-plane sensitive tip. The Co adatoms adsorb at the hollow sites of the locally nearly antiferromagnetic c(2x2) unit cell (see Fig. 1(a) in the main article) and couple ferromagnetically to the nearest underlying Mn atoms[1]. The orientation of the magnetic moments of Co adatoms adsorbed at hollow sites of neighboring rows within the spin spiral (black box in Supplementary Fig. 1) differs by an angle of about 173°. It was recently shown that the appearance and symmetry of the Co adatoms, as observed in SP-STM, strongly depends on the orientation of their magnetic moments with respect to the magnetization of the tip[2]. For a parallel out-of-plane alignment between the magnetic moments of the tip and the Co adatom, an oval protrusion is observed in SP-STM images, while the Co adatom appears as a dumbbell shape along $[1\bar{1}0]$ for an anti-parallel out-of-plane alignment[1]. For magnetic tips with an in-plane magnetization, the appearance of the Co adatom in SP-STM images is again significantly different[2]. We use of these different appearances of Co adatoms in SP-STM images to characterize the magnetic sensitivity of the tip. We laterally manipulated at least two Co adatoms (manipulation parameters V_s = -2 mV and I_T = -30 nA) to neighboring atomic hollow-site rows along $[1\bar{1}0]$ at the location with maximum contrast of the spin-spiral period. We monitored the appearances of the Co adatoms in our SP-STM, and compared them to the expected shapes and symmetries[2]. We

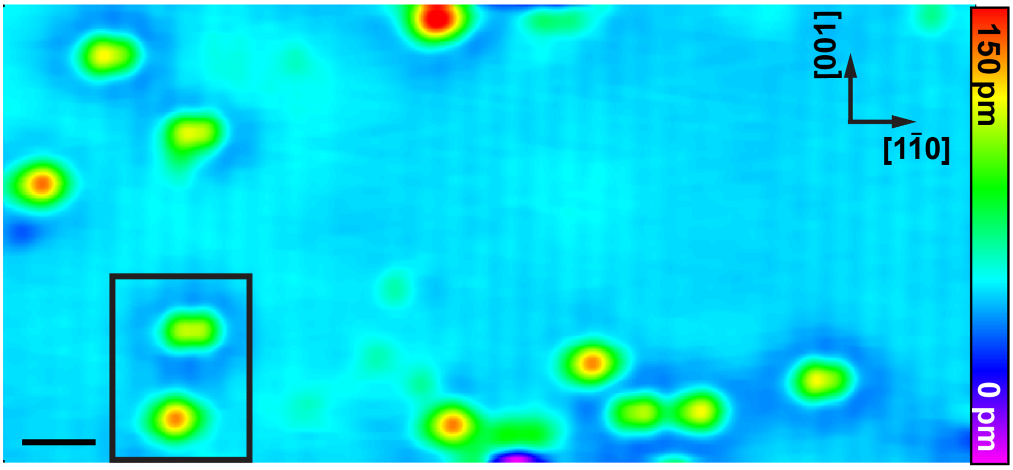

Supplementary Figure 1: SP-STM overview image of the Mn monolayer on W(110). The SP-STM image (V_s = -10 mV, I_T^{cc} = -2 nA) was acquired with a mainly out-of-plane sensitive magnetic tip. Black box: Deposited Co adatoms adsorbed onto two neighboring hollow-site rows (h_i and h'_i) within the c(2x2) unit cell (see Fig. 1(a) in the main article). The scale bar corresponds to 1 nm.

continued with gentle dipping of the tip into the Mn layer, as described above, until we observed the expected appearances for an out-of-plane magnetization of the tip. To make sure that we have achieved out-of-plane magnetic sensitivity, we moved the Co adatoms afterward by one lattice constant and monitored if we see the expected change of their appearance in SP-STM images. Using this procedure, we can ensure that all tips used for the SPEX measurements have a dominant out-of-plane magnetic sensitivity. However, we cannot exclude a small misalignment of the magnetization of the tip from the out-of-plane direction that is smaller than the difference between the magnetic moments of Mn atoms in neighboring rows of the spin spiral (~173°). For that, an external magnetic field would be required to provide a better magnetic alignment.

Supplementary Note 2: Experimental details of constant-height imaging and raw data

Prior to the acquisition of constant-height images, an overview constant-current image (Supplementary Fig. 1) was always measured in order to ensure that the area is atomically flat and no Co adatoms or other adsorbates as well as defects is present within at least 1 nm around the area of interest of the spin spiral. In case of only Co adatoms present in the area, they were moved away by lateral manipulation. In order to reduce the creep from the piezo scanner, repeated close-up constant-current images at setpoint V_s = -10 mV and I_T^{cc} = -2 nA with z_{mod} = 50 pm were acquired until no lateral creep could be discerned anymore between the images. We used the Δf values at this feedback setpoint to characterize the bluntness of our tips, which were typically between -10 Hz and -17 Hz). Afterwards, the current feedback loop was switched off at z_0 at the position of parallel alignment between the magnetization of the tip and the Mn spin spiral (cross in Supplementary Fig. 2(a)). V_s was reduced to about $|V_s|$ = 0.1 mV, and the tip was approached toward the surface by -0.29 nm (corresponding to z_1) at which the constant-height imaging was started with a scan speed of 0.35 nm/s. While scanning, we recorded the constant-height frequency shift Δf^{ch}, the constant-height current I_T^{ch}, as well as the oscillation amplitude z_{mod} and the excitation voltage V_{ex}. In certain cases, we did not determine the tilt angle of

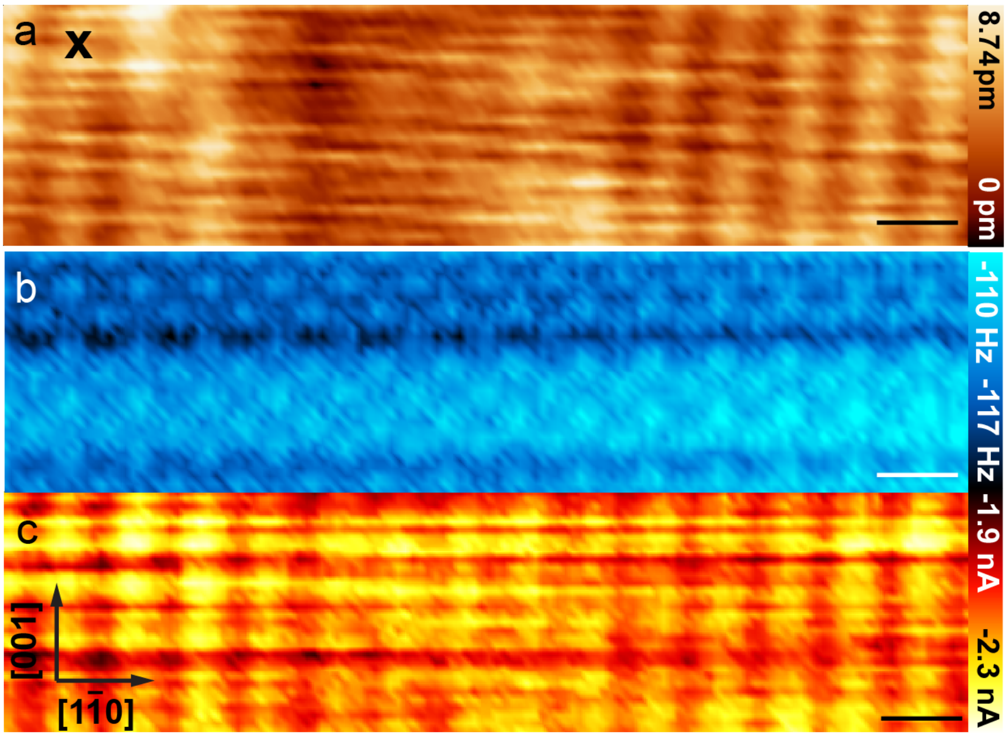

Supplementary Figure 2: Raw data constant height imaging. (a) Constant-current SP-STM image, taken prior to acquiring the constant-height image in Supplementary Fig. 1 of the main text. Parameters: V_s = -10 mV and I_T^{cc} = -2 nA, out-of-plane magnetic sensitive tip. The image was processed by a global plane fit and median filter by 3 points in x- and 3 points in y-direction. (b,c) Raw data of Supplementary Fig. 1 of the main text: constant-height frequency shift Δf^{ch} (b), and constant-height current I_T^{ch} (c). Parameters: z_{mod} = 50 pm, V_s = -0.01 mV, out-of-plane magnetic sensitive tip. The current feedback loop was opened at V_s = -10 mV and I_T^{cc} = -2 nA at the position marked by a cross in (a) before the tip was brought closer by -0.29 nm for (b) and (c). The scale bars in (a) to (c) corresponds to 0.5 nm.

the surface with respect to the tip precisely enough. This misalignment results in a linear change of Δf^{ch} with respect to, *e.g.*, the [1$\bar{1}$0] direction of a few Hz at z_1 between $\varphi = 0°$ and $\varphi = 180°$ of the spin spiral (see Supplementary Fig. 2(b)). However, on top of this linear offset, we observe variations of the magnitude of Δf^{ch} reflecting the chemical interaction of the tip with the Mn atoms as well as the magnetic exchange interaction, due to the reversal of the out-of-plane projection of the surface magnetization within the spin spiral (see main article Fig. 1(c) and Supplementary Fig. 2(b)).

Supplementary Note 3: Assignments of top and hollow sites

It is not straightforward to determine the top and hollow sites from the contrast in constant-height Δf images that reflect the averaged force gradient at a fixed tip-sample distance. Instead, we compare the total measured $F(\Delta z)$ at different sites within the c(2x2) unit cell with calculated force-distance curves.

Supplementary Fig. 3 shows plots of the calculated total energy of the system and total force acting on the tip for a Mn-terminated tip along the [110] (i.e. *z*) direction as a function of tip-sample separation (*Δd*) for top (T) and hollow (H) sites. For both parallel (P) and antiparallel (AP) alignment of the magnetic moments of the tip and the Mn atoms (see Supplementary Note 7 for more details), the absolute values of the total forces on the top sites are smaller than on the hollow sites. The same trend is observed in the experiment (Supplementary Fig. 4(b)): *F*(*Δz*) curves acquired at sites within the c(2x2) unit cell that appear as protrusions in constant-height *Δf* images (main article Fig. 1(c) and Supplementary Fig. 2(b)), have smaller absolute force values than those measured at sites that appear as depressions. Therefore, we assign the protrusions and depressions in constant-height *Δf* images to the top and hollow sites of the c(2x2) unit cell, respectively.

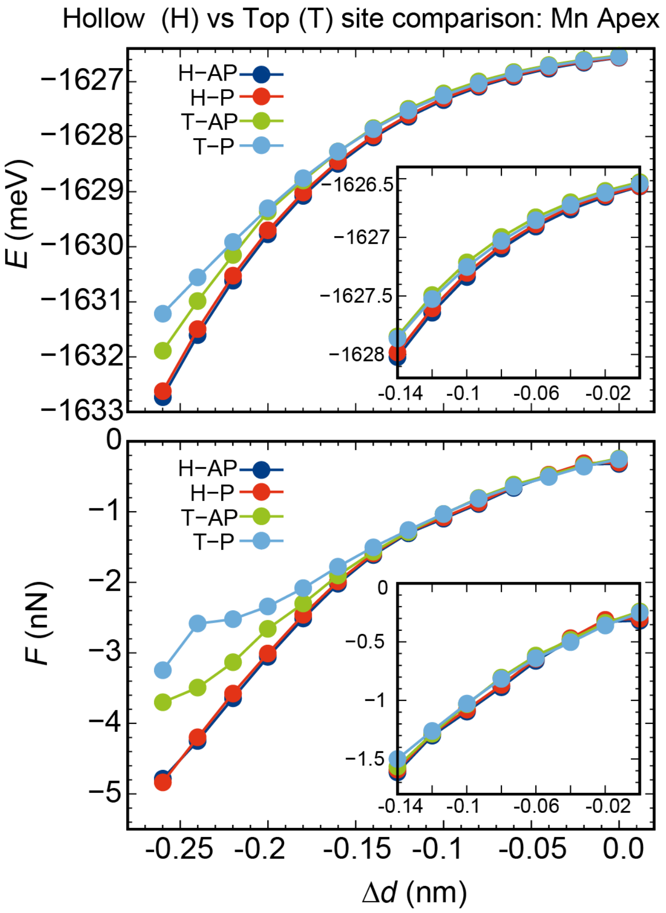

Supplementary Figure 3: DFT calculation of the total energy and forces. Calculated DFT total energy (upper panel) of the system and total forces (lower panel) acting on the tip with an Mn-terminated Fe tip along the [110] (*z*) direction as a function of the change of the tip-sample separation (*Δd*), shown on top (T) and hollow (H) sites for both parallel (P) and anti-parallel (AP) alignment of surface Mn and tip-apex spin. (For computational details see Supplementary Note 7). *Δd* is defined as $d–d_0$, where d_0 = 0.5 nm and *d* is the tip-sample. Separation (cf. Supplementary Fig. 8).

Supplementary Note 4: Details on distance-dependent measurements

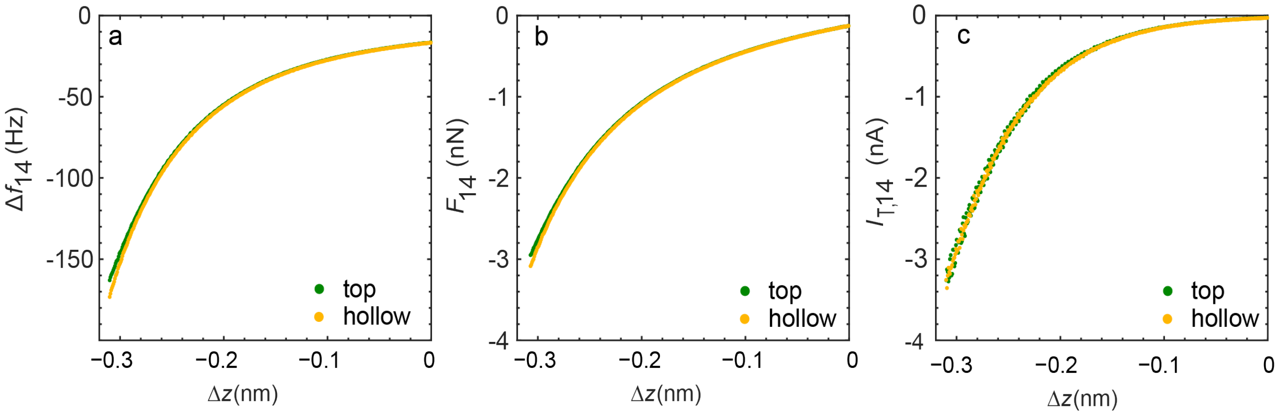

Supplementary Figure 4: Raw data of distant-dependent measurements. Distance-dependent measurement acquired on the top (t_{14}) and hollow (h_{14}) site (see main article Fig. 1(d) for definition). (a) Raw-data frequency shift Δf_{14}, (b) total force F_{14} calculated from Δf_{14}, and (c) raw-data current, $I_{T,14}$ versus the tip displacement Δz. The position Δz = 0 nm corresponds to z_0 and negative Δz reflects smaller tip-surface distances. Parameters: oscillation amplitude z_{mod} = 50 pm, V_{s} = -0.01 mV, out-of-plane magnetic sensitive tip. The current feedback loop was opened at V_{s} = -10 mV and $I_{\mathrm{T}}^{\mathrm{cc}}$ = -2 nA, corresponding to Δz = 0 nm.

In order to quantify the forces between the tip and the spin spiral, distance-dependent measurements on the top (t_i and t'_i) and hollow (h_i and h'_i) sites of the c(2x2) unit cell were performed. First, a constant-height image with atomic resolution of the spin spiral was obtained to resolve the top and hollow sites of the c(2x2) unit cell (Supplementary Note 2 and Supplementary Fig. 2). The tip-sample distance was increased to z_{s} (where $z_{\mathrm{s}} = z_0$ + 50 pm) and the tip was moved laterally to the measurement position. Then, the tip was brought closer to and subsequently retreated from the surface by $\Delta z \leq 360$ pm in steps of ~ 0.8 pm while recoding the frequency shift Δf, the current I_T, as well as the oscillation amplitude z_{mod} and the excitation voltage V_{ex} at each step for 50 to 100 ms. To account for possible lateral piezo creep, we repeatedly acquired data in an alternating pattern at t_i/t'_i and h_i/h'_i for three to five times, and averaged the data at equivalent positions. To exclude vertical piezo creep, we compared the frequency shift Δf_0 at z_0 before and after measurements at the four atomic sites (t_i, t'_i, h_i, h'_i). If the variation of Δf_0 was smaller than the noise in Δf (i.e. ±0.1 Hz), we considered the vertical creep to be negligible. In addition, we averaged the data from the forward and backward sweeps. The total force F (Δz) was extracted from Δf (Δz) by utilizing the formula in ref. [3]:

$$F(\Delta z) = 2k\int_{\Delta z}^{\infty}\left(1+\frac{z_{mod}^{1/2}}{8\sqrt{\pi(t-\Delta z)}}\right)\frac{\Delta f(t)}{f_0} - \frac{z_{mod}^{3/2}}{\sqrt{2(t-\Delta z)}}\frac{d}{dt}\left(\frac{\Delta f(t)}{f_0}\right)dt$$

with z_{mod} = 50 pm, f_0 = 30.8 kHz the resonance frequency and k = 1800 N/m the stiffness of the qPlus sensor[4]. Supplementary Fig. 4 shows the raw data ($\Delta f_{14}, F_{14}, I_{T,14}$) for the data shown in the main article Fig. 2 at t_{14} and h_{14}. Note that the total forces (Supplementary Fig. 4(b)) range up to a few nN. We observe a clear difference in the total forces on the different atomic sites (t_{14} and h_{14}). We refer to Supplementary Note 3 for the definition of the top and hollow sites. Interestingly, no clear difference in the currents can be resolved (Supplementary Fig. 4(c)). Our data shows that the forces are more sensitive to the atomic-scale configuration than the current.

Supplementary Note 5: Distance-dependent measurements along half the spin spiral

We perform MExFIS along half a period of the spin spiral as sketched in Supplementary Fig. 5(a). Prior to the data acquisition, the tip magnetization was prepared to have dominantly out-of-plane magnetization using the procedure described in Supplementary Note 1. Then, $\Delta f(\Delta z)$ and $I_T(\Delta z)$ were measured at the hollow sites (h_i and h'_i) along the spin spiral. We made sure that the acquired data does not indicate artifacts according to our definitions in Supplementary Note 6. Supplementary Fig. 6 shows Δf_{zc}, F_{zc}, and $I_{T,zc}$ averaged within a distance range $z_c = ((z_0 - 0.27) \pm 0.1)$ nm versus the site i within the spin spiral for h and h'. We observe a linear decrease in magnitude of $\Delta f_{zc}(i)$, $F_{zc}(i)$, and $I_{T,zc}(i)$ which we assign to a small tilt of the surface that we did not entirely compensate for. Nevertheless, we observe clear differences in the magnitudes of $\Delta f_{zc}(i)$, $F_{zc}(i)$, and $I_{T,zc}(i)$ between h_i and h'_i, which reflects the different spin alignments within the spin spiral. For the discussion of the magnetic contribution to the force, we focus on $F_{ex,i}(\Delta z)$, derived from $\Delta f_{ex,i}$, and $A_i(\Delta z)$ (Supplementary Fig. 5(b,c)) as well as $F_{ex,zc}(i)$ and $A_{zc}(i)$ (Supplementary Fig. 5(d,e)). We observe the expected gradual decrease of the absolute values for $F_{ex,zc}(i)$ and $A_{zc}(i)$ from i = 1 and i = 7 (Supplementary Fig. 5(d,e)). In addition, $F_{ex,zc}(1)$ exhibits a different sign than $F_{ex,zc}(14)$, which is expected considering the reversal of the magnetic moments of the spin spiral between $\varphi = 0°$ and $\varphi = 180°$. However, we observe indications that the magnetization of the tip has changed during the data acquisition. There is an abrupt

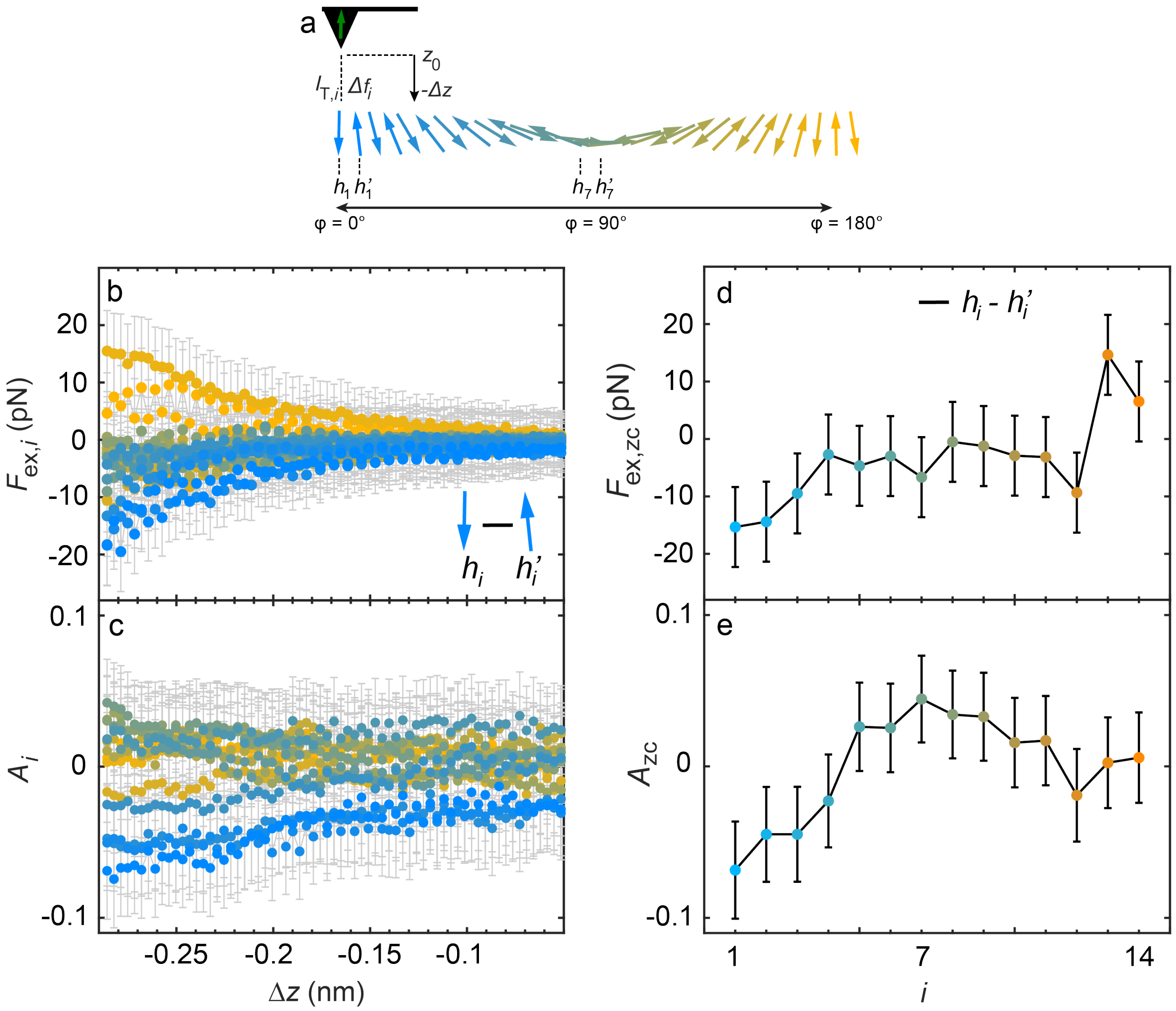

Supplementary Figure 5: Distance-dependent measurements along half the spin spiral. (a) Sketch of the cycloidal spin spiral for the hollow sites and illustration of the distance-dependent measurement scheme. (b) Magnetic exchange force $F_{ex,i}(\Delta z)$ derived from $\Delta f_{ex,i}(\Delta z)$, with $\Delta f_{ex,i}(\Delta z) = \Delta f_i(\Delta z) - \Delta f'_i(\Delta z)$, versus the tip displacement Δz for different hollow sites h_i/h'_i. (c) Current asymmetry $A_i(\Delta z) = \frac{I_{T,i}(\Delta z) - I'_{T,i}(\Delta z)}{I_{T,i}(\Delta z) + I'_{T,i}(\Delta z)}$. The position $\Delta z = 0$ nm corresponds to z_0 and negative Δz reflects smaller tip-surface distances. The grey error margins reflect an uncertainty of ±0.2 Hz and ±3% for $\Delta f_i/\Delta f'_i$ and $I_{T,i}/I'_{T,i}$, respectively. (d,e) $F_{ex,zc}$ and A_{zc} versus the hollow site position i within the spin spiral at averaged within $z_c = ((z_0 - 0.27) \pm 0.1)$ nm. Parameters: oscillation amplitude $z_{mod} = 43$ pm, $V_s = -0.1$ mV. The tip magnetization during the data acquisition may differ from the characterized out-of-plane direction prior and after the distance-dependent measurements.

change between $F_{ex,zc}(12)$ and $F_{ex,zc}(13)$, which we interpret as a change of the magnetization of the tip due to large magnetic exchange interactions with the surface and the softness of the magnetic tip. Furthermore, a few curves show a behavior as characterized by the tip type in Supplementary Fig. 7(a), in addition to the tip type Supplementary Fig. 7(d). Therefore, we excluded the data for $i = 7$ to $i = 14$ from our discussion. To avoid spontaneous changes of the tip magnetization, magnetic tips with a larger magnetic remanence are required.

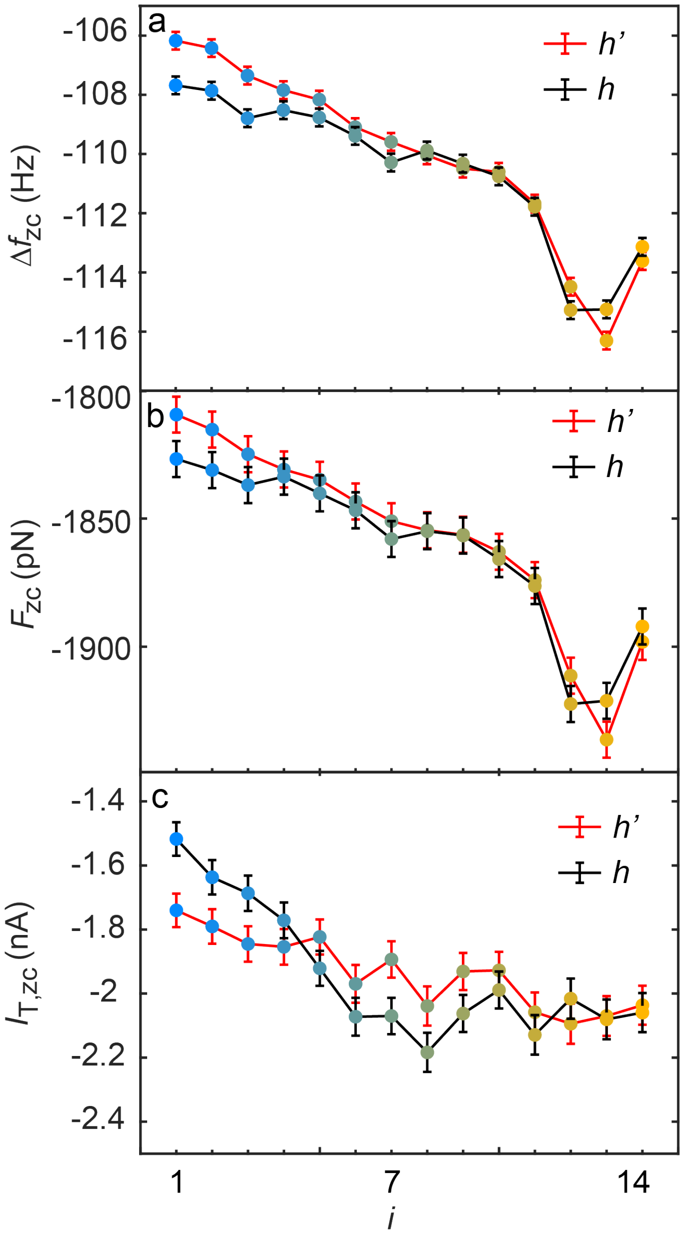

Supplementary Figure 6: Data along half the spin spiral at a fixed tip-surface distance. (a) Δf_{zc}, (b) F_{zc}, and (c) $I_{T,zc}$ versus i at z_c for different hollow sites h/h'. Parameters: z_{mod} = 43 pm, V_s = -0.1 mV. The tip magnetization during the data acquisition may differ from the characterized out-of-plane direction prior and after the distance-dependent measurements.

Supplementary Note 6: Characterization of different tip types

During our distance-dependent measurements, we have observed various different tip behaviors. In total, we have used 38 different atomic-scale tips (out-of-plane sensitive tips: 7) which we categorized into different classes by analyzing $\Delta f(\Delta z)$, $I_T(\Delta z)$ and $V_{ex}(\Delta z)$ (Supplementary Fig. 7). A stable tip behavior is demonstrated in Supplementary Fig. 7(d) where no changes in the forward and backward

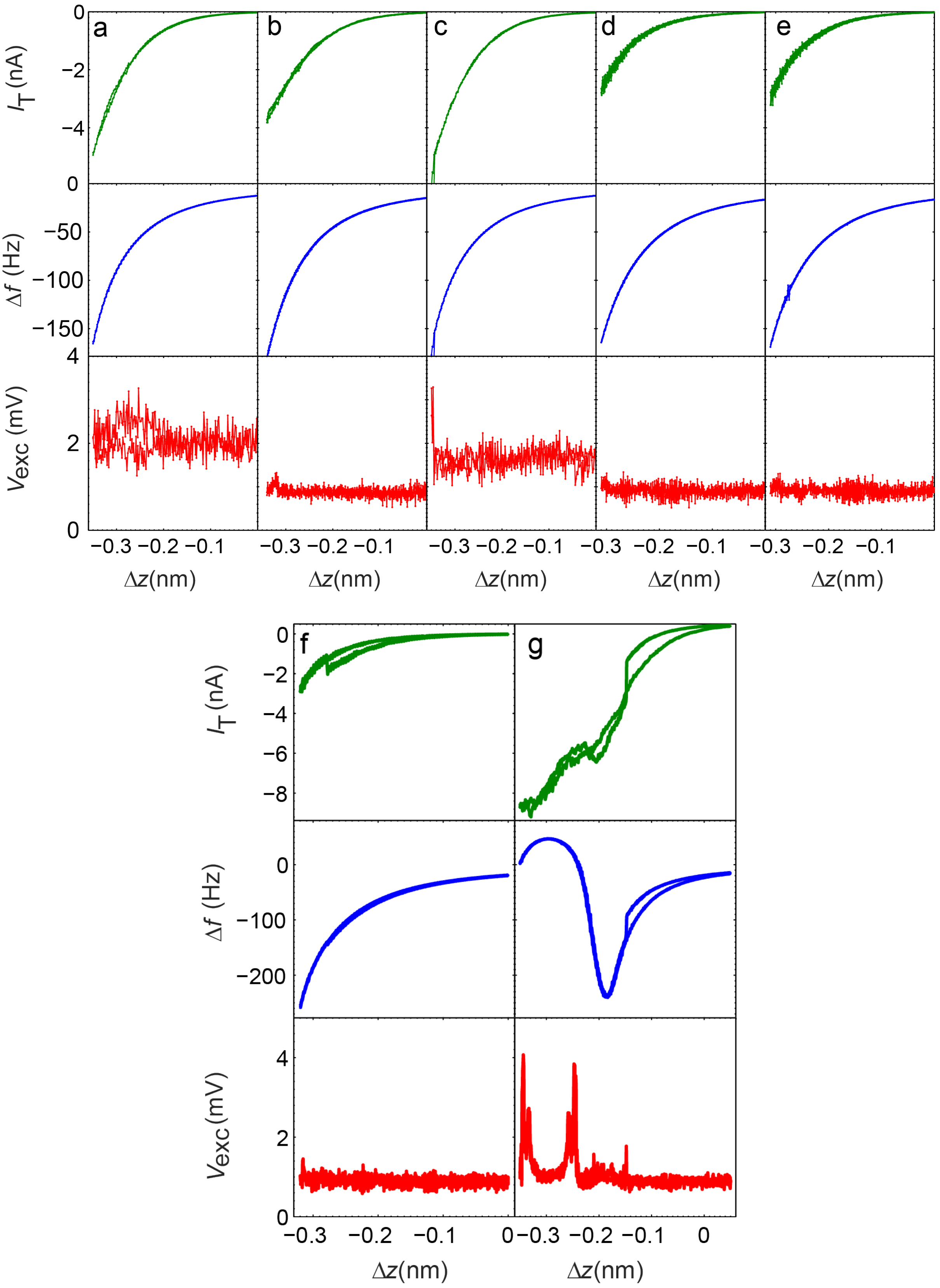

Supplementary Figure 7: Characterization of the different observed tip types. Each panel ((a) to (g)) shows the frequency shift Δf, the current I_T and the excitation voltage V_{ex} with respect to the tip displacement Δz for a different observed tip type. Parameters: oscillation amplitude z_{mod} = 50 pm, V_s = -0.01 mV, tip magnetization normal to the surface. The current feedback loop was opened at V_s = -10 mV and I_T^{cc} = -2 nA corresponding to Δz = 0 nm.

sweeps are observed. This indicates that both the geometric structure and the magnetization of the tip were stable. In Supplementary Figs. 7(a) and (b), the $\Delta f\,(\Delta z)$ curves show a smooth behavior, however, a small hysteresis between forward and backward sweeps of $I_T(\Delta z)$ and $V_{ex}(\Delta z)$ for Δz < -0.2 nm is present for the tip in Supplementary Fig. 7(a), as well as a small peak in $V_{ex}(\Delta z)$ at Δz = -320pm in Supplementary Fig. 7(b). We interpret the variations in $V_{ex}(\Delta z)$ as the presence of inelastic processes. However, SP-STM images acquired afterwards do no indicate a change of the magnetization nor a change of the geometric structure of the tip. We therefore attribute these findings to small fluctuations of the tip magnetization at small tip-sample distances. For the curves in Supplementary Fig. 7(c), the slope of $\Delta f\,(\Delta z)$ and $I_T\,(\Delta z)$ changes drastically for Δz < -340 pm, together with a sudden increase in $V_{ex}\,(\Delta z)$. We interpret these observations as structural relaxations between the tip and the surface, probably due to an adsorbate at the tip apex. In a few occasions, we observed a short spike in $\Delta f(\Delta z)$ which we assign to vibrational/acoustic noise of the cryogenic UHV system (Supplementary Fig. 7(e)). Examples of obviously irreversible tip changes are shown in Supplementary Figs. 7(f) and 7(g), as either a clear jump in $I_T(\Delta z)$ or irreversible changes in all signals, suggesting a dramatic change not only in the magnetization but also in the geometric structure. In our discussion of distance-dependent magnetic exchange force and spin polarization in the main article Figs. 2, we show data acquired with tips as defined in Supplementary Fig. 7(d). The data in Fig. 3 of the main article shows characteristics as defined in Supplementary Figs. 7(a) and 7(d). In order to avoid fluctuations of the tip magnetization, as well as a misalignment from the out-of-plane magnetization (see Supplementary Note 1), an external magnetic field would be required.

Supplementary Note 7: Details on the tip and sample geometry for the DFT calculation

For the combined system, we have considered two different positions of the tip: (i) top position, where the apex atom is on top of a Mn atom and (ii) hollow position, where the tip apex atom is on top of a hollow site (Supplementary Fig. 8). For both top and hollow positions, we carried out two sets of

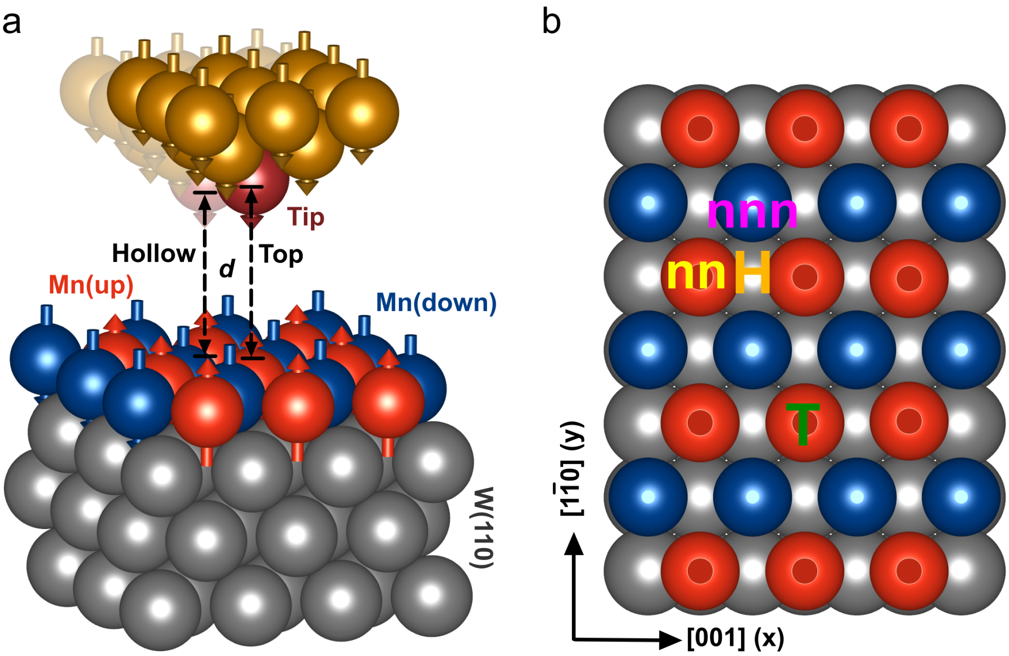

Supplementary Figure 8: Tip and sample geometry for the DFT calculation. (a) Perspective view of the Fe cluster tip approaching the Mn monolayer on W(110) for an antiparallel alignment of the magnetic moments of the apex tip atom and the Mn surface atom underneath (top site). For hollow site, the antiparallel alignment of the magnetic moments is in between the apex tip atom and the nearest-neighbor Mn surface atoms (nn). The approximated collinear antiferromagnetic arrangement of the Mn magnetic moments in the monolayer is indicated with the red/blue arrows. The maroon color ball indicates the apex tip atom. We have considered three different apex tip atoms in our calculation (Fe, Co, and Mn). *d* denotes the distance between the centers of the tip apex atom and the surface Mn atom underneath, not considering relaxations due to the interaction. The arrows indicate the direction of the magnetic moments of the atoms. (b) Top view of the c(6×6) antiferromagnetic surface unit cell considered in our calculation to model the Mn/W(110) surface. Red and blue spheres denote the Mn atoms with up and down magnetic moment direction, respectively. ‘T’ and ‘H’ represent the top and hollow site on the Mn surface, respectively. ‘nn’ and ‘nnn’ are the nearest-neighbor and next-nearest neighbor Mn surface atoms with respect to the hollow site.

calculations, one for a parallel (*p*) alignment of the magnetic moments and the other for an antiparallel (*ap*) alignment of the magnetic moments. For the top position, the *p* and *ap* alignment of the magnetic moments are defined with respect to the magnetic moments of tip apex atom and the surface Mn atom underneath. In case of the hollow sites, the *p* and *ap* alignments were defined with respect to the magnetic moments of the nearest neighbor (nn) surface Mn atoms (cf. Supplementary Fig. 8(b)). The tip was approached toward the surface through a trajectory of discrete points, and for every point we carried out a DFT calculation to obtain the energy and forces acting on the system. We have used a symmetric slab configuration to model the Mn/W(110) surface and therefore placed the tip on each side of the symmetric slab. We have used a 50 Å tall supercell for our calculations in order to ensure

a vacuum of at least 20 Å along the vertical direction between the base of tips in two adjacent unit cells.

Supplementary Note 8: Impact of structural relaxations

The left panel of the Supplementary Fig. 9 shows the relative tip apex displacement (Fe apex tip) compared to the unrelaxed position due to tip-surface interaction. As expected, the displacement is larger at the hollow site as the tip apex atom has much space to move along the *z*-direction compared to the top position. The right panel of Supplementary Fig. 9 shows the comparison of the exchange energy and the exchange force before and after the geometry relaxation due to tip-sample interaction (Fe apex tip). Our results indicate that the qualitative behavior of the exchange energy and the

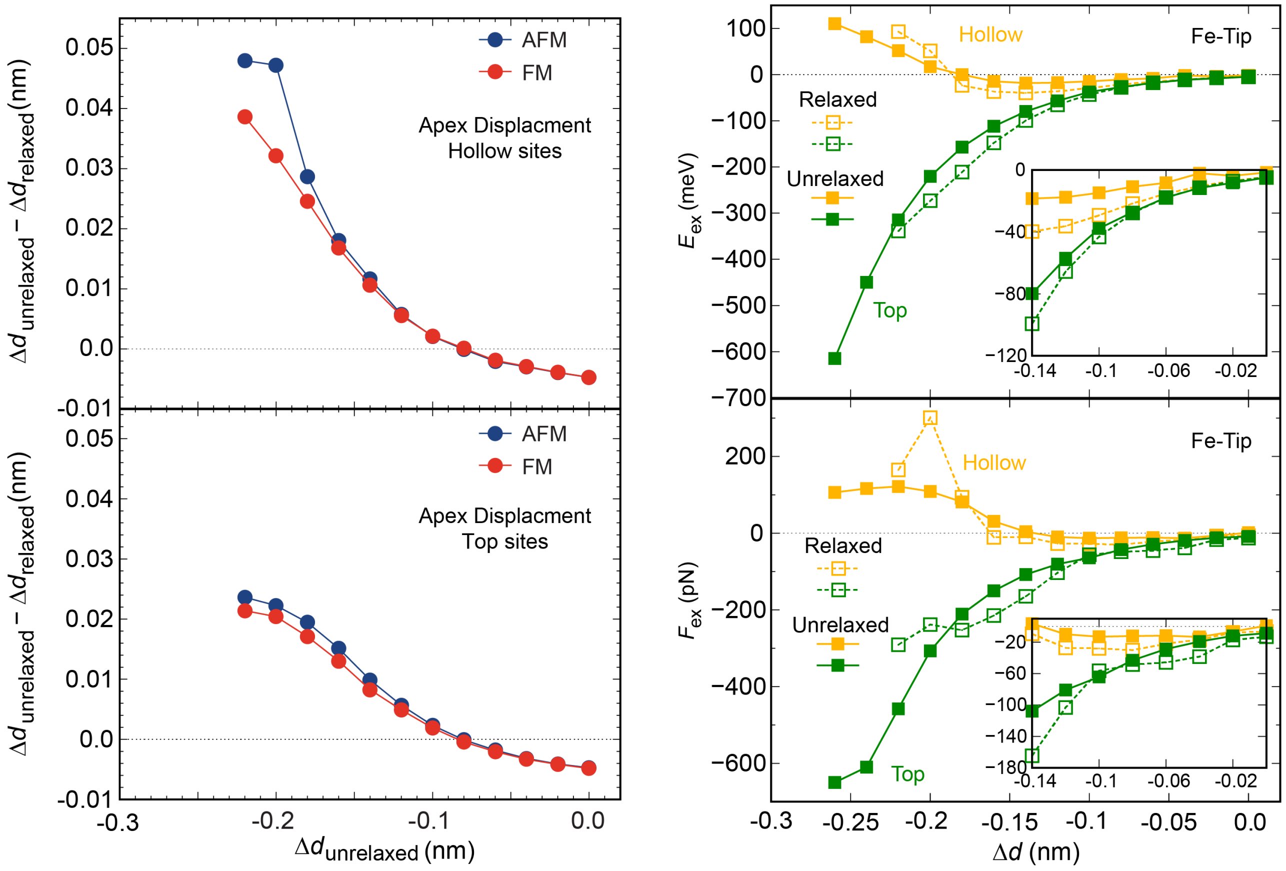

Supplementary Figure 9: Calculated structural relaxations. (Left panel) Relative Fe tip apex atom displacement after geometry relaxation due to tip-sample interaction. (Right panel) Comparison of the exchange energy and the exchange force before and after the geometry relaxation due to tip-sample interaction. Δd is defined as $d - d_0$, where d_0 = 0.5 nm and d is the tip-sample separation (cf. Supplementary Fig. 8).

exchange force will remain similar even after we consider geometry relaxation due to tip-sample interaction.

Supplementary Note 9: Charge density difference plots

We have calculated the spin-resolved charge density difference $\Delta\rho$ at a change of tip-sample distance of $\Delta d = -0.1$ nm (see Supplementary Fig. 10, with $\Delta d = d - d_0$, where $d_0 = 0.5$ nm and d is the tip-sample separation) in order to understand the origin of the exchange interaction between the tip and the sample. The charge density differences are calculated using the following formula

$$\Delta\rho = \rho^{\mathrm{tip+sample}} - \rho^{\mathrm{tip}} - \rho^{\mathrm{sample}} \quad , \qquad (1)$$

where $\rho^{\mathrm{tip+sample}}$ denotes the charge density of the coupled system while ρ^{tip} and ρ^{sample} are calculated for the isolated tip and Mn/W(110) surface, respectively. Here, we focus on Fe- and Mn-terminated tips. The Co terminated tip is similar to the case of Fe. At the chosen tip-sample distance of $\Delta d = -0.1$ nm, the sign of the exchange interactions is opposite for these two tips. If we look at the antiparallel configurations for both the top and hollow sites, we find that the charge redistribution at the tip apex and probed surface atoms appears very similar for both the Fe- and Mn-terminated tip. The plots for the majority and the minority channels are similar to each other with a reversal of charge accumulation and depletion at the tip apex atom and the interacting surface for both channels. This kind of charge distribution is the characteristic for a short-range direct exchange mechanism between the *d* orbitals due to the formation of spin-dependent covalent bonds[7, 8] which favor antiferromagnetic coupling.

In the parallel magnetic configuration, the characteristic of $\Delta\rho$ is different in the two spin channels. In the majority spin channel, we notice a charge depletion at the apex and surface atoms and an accumulation in between them, for both tip terminations. This charge redistribution can be explained based on the indirect Zener double exchange mechanism[9, 10] which leads to ferromagnetic exchange coupling between tip and sample[8]. The origin of this mechanism is due to delocalized *sp*-conduction

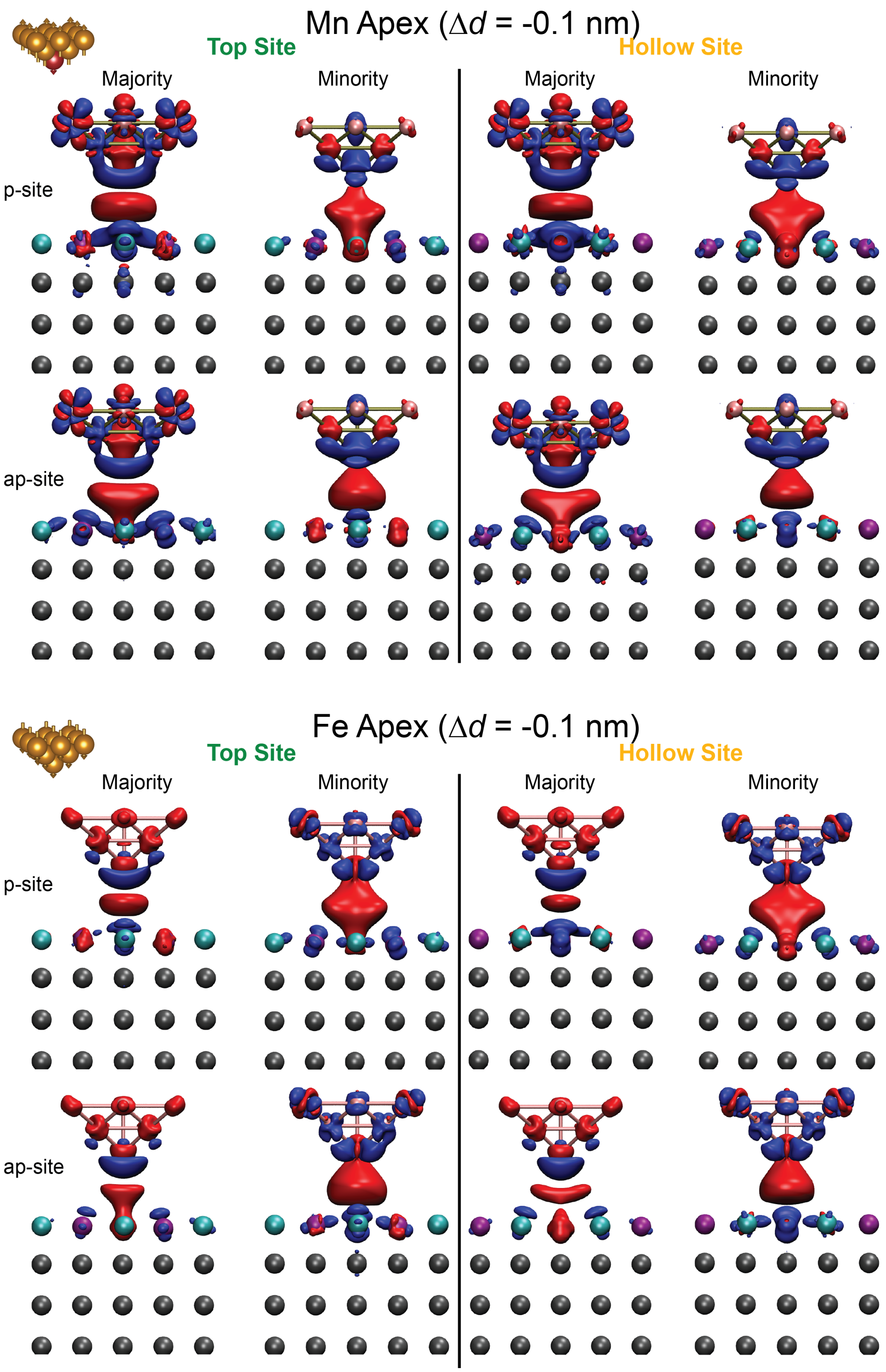

Supplementary Figure 10: Spin-resolved charge-density difference plots. The panels show the spin-resolved charge-density difference plots along the $[1\bar{1}0]$ direction for the interaction of Mn- and Fe-terminated tips with the Mn monolayer on W(110) at a tip-sample separation of $\Delta d = -0.1$ nm ($\Delta d = d - d_0$, where d_0 = 0.5 nm and d is the tip-sample separation, cf. Supplementary Fig. 8). The configurations of antiparallel (*ap*) and parallel (*p*) alignments between the magnetic moments of the tip apex and surface atoms are displayed as indicated at the left of each panel. The plots are obtained from the difference of charge densities ($\Delta\rho$) of isolated and interacting tip and sample (i.e., $\Delta\rho = \rho^{tip+sample} - \rho^{tip} - \rho^{sample}$) for the majority and minority spin channels (indicated at the top of each panel). The results presented here correspond to unrelaxed geometries for tip and sample due to their interaction.

electrons which couple with the *d* states. However, in the minority channel, the charge density is depleted at the apex atom and accumulated at the probed surface which is the characteristic for a direct exchange mechanism between the *d* orbitals due to the formation of spin-dependent covalent bonds[7]. For a Mn-terminated tip, the double exchange mechanism is stronger than the direct antiferromagnetic exchange due to the larger *s*-*d* coupling. Hence the total exchange interaction is always ferromagnetic for all tip-sample separations in the case of hollow sites and for larger tip-sample separations in the case of top sites. The coupling changes to an antiferromagnetic coupling at close tip-sample separation. For a Fe-terminated tip, the Zener type exchange mechanism is weaker compared to direct *d*-*d* exchange due to a lower *s*-*d* coupling[9, 10, 11], hence we observe an overall antiferromagnetic exchange at the top sites. For the hollow site, this is much weaker at large tip-sample separation and changes to a ferromagnetic exchange at close tip-sample separation.

Supplementary Note 10: Tip-dependent variations of the magnetic exchange force and spin polarization

The out-of-plane magnetic sensitive tips were prepared by gently dipping the bulk Fe tip into Mn monolayer with adsorbed Co-adatoms. Therefore, in addition to different geometric atomic-scale structures of the tip, the apex may also have a different chemical termination (Fe, Mn, Co). Supplementary Fig. 11 shows the tip-dependent variations of the magnetic contribution to the frequency shift (Δf_{ex}), the magnetic exchange force (F_{ex}) and the current asymmetry (A) that we relate to the spin polarization, versus Δz at the positions i = 1 and i = 14 of the spin spiral as defined in the main article Fig. 1(d). Please note that Supplementary Fig. 11 only shows data acquired with tips that have a dominantly out-of-plane magnetic sensitivity according to our characterization in Supplementary Note 1, and belong to the tip types as defined in Supplementary Fig. 7 (b,d). While

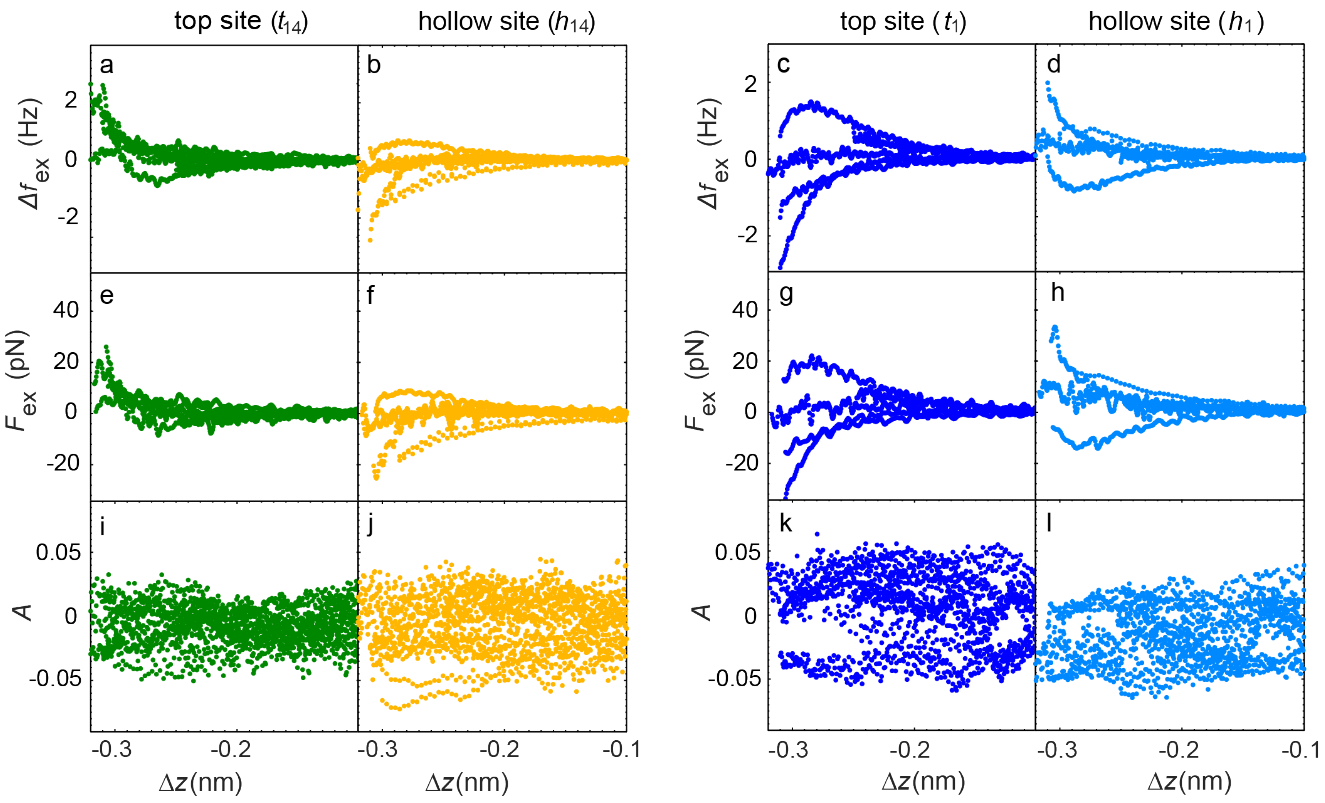

Supplementary Figure 11: Distance-dependent data acquired with the tip types as characterized in the Supplementary Figs. 7(a) and 7(d). Measured magnetic contribution to (a-d) the frequency shift $\Delta f_{ex}(\Delta z)$, (e-h) derived magnetic exchange force $F_{ex}(\Delta z)$, and (i-l) current asymmetry $A(\Delta z)$ versus the tip displacement (Δz) for different dominantly out-of-plane magnetic sensitive tips at hollow (h_1 and h_{14}) and top (t_1 and t_{14}) sites within the c(2x2) unit cell. For all datasets: oscillation amplitude $z_{mod} = 50$ pm, $|V_s| \leq 0.05$ mV. The position $\Delta z = 0$ nm corresponds to z_0 and negative Δz reflects smaller tip-surface distances.

$A(\Delta z)$ (Supplementary Figs. 11(i-l)) does not show a strong variation for different tips, there are various different trends present for $F_{ex}(\Delta z)$ (Supplementary Figs. 11(e-h). This may be explained by different chemical tip terminations which are out of the scope of our tip characterization methods described in Supplementary Note 1 and Supplementary Note 6. As our calculations show (Fig. 4 of the main article), the magnetic exchange forces are highly sensitive to not only the atomic site (top or hollow) but also to the chemical tip termination. This is in agreement with the strong variation of $F_{ex}(\Delta z)$ for mainly out-of-plane magnetic sensitive tips observed in Supplementary Fig. 11. The absence of variations of $A(\Delta z)$ indicates that the spin polarization is less sensitive to the chemical tip termination and the resulting different orbital composition of the tip.

Supplementary Note 11: Spin polarization

The spin polarization of the tunneling current found on Mn/W(110) is unexpectedly small (see Fig. 2 in the main article). Our DFT calculation shows a spin polarization value of ~20% of the vacuum LDOS in an energy range of ± 0.2 eV near the Fermi energy for Mn/W(110) (Supplementary Fig. 12). Supplementary Fig. 13 shows the LDOS above the Mn/W(110) surface in comparison with the orbitally decomposed LDOS at the Mn atom underneath. We find that the peaks in the vacuum LDOS stem from p_z states and that there are considerable contributions from spin-up and -down states leading to an overall spin polarization of about 20% in the vicinity of the Fermi energy. The *d* states of the Mn atoms are pushed far away from the Fermi energy due to the large exchange splitting resulting in a large magnetic moment [5]. This is different from Fe where we have a considerable contribution from the minority *d* states close to the Fermi energy[6]. In particular, the d_{z^2} states are energetically relatively far away from the Fermi energy for Mn/W(110). In general, a 3*d* material with a very large magnetic moment, such as Mn, will have a smaller spin polarization in the vicinity of the Fermi energy while

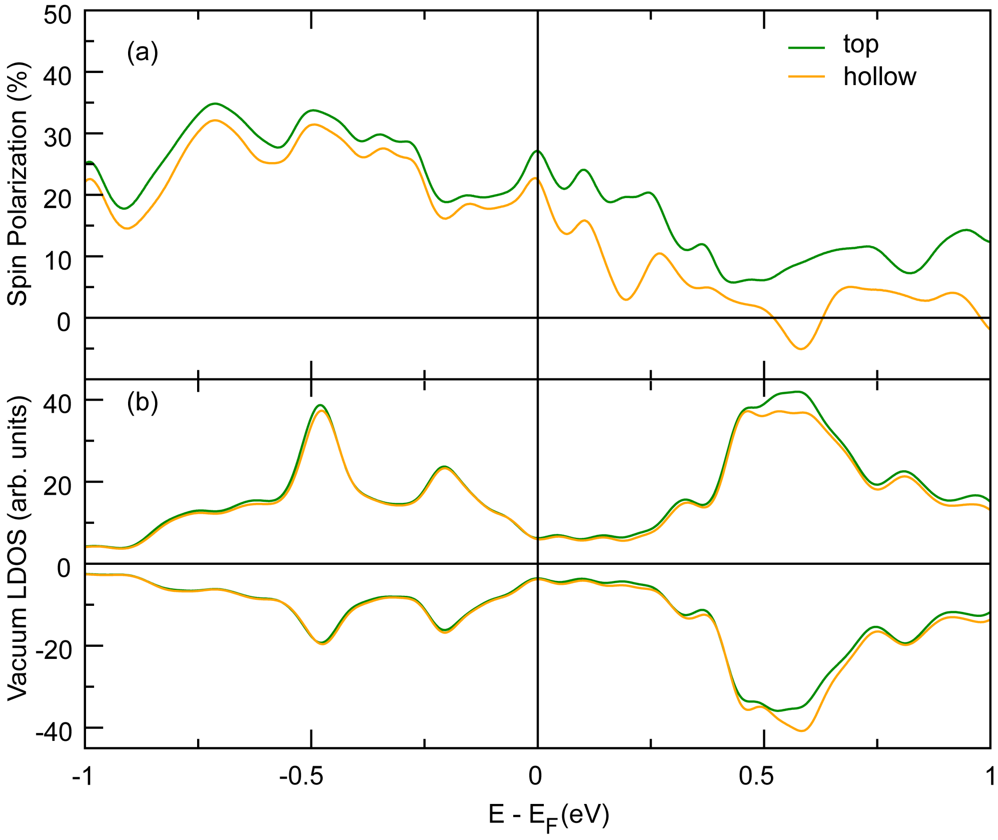

Supplementary Figure 12: Comparison of spin polarization and vacuum LDOS. (a) Spin polarization and (b) vacuum LDOS at 5 Å above the Mn/W(110) surface for top (green) and hollow (orange) sites.

the large exchange interaction with a magnetic tip is not affected. Supplementary Fig. 12 shows a comparison of spin polarization and vacuum LDOS 5 Å above the Mn surface for top (green) and hollow (orange) sites. From the figure, one can clearly observe that the vacuum LDOS does not change significantly between top and hollow sites as found in the experiments (cf. Fig. 2(f,g) in the main article). The calculated spin polarization is ~ 20% for the top site near the Fermi energy. This value is lower by 5% in the case of the hollow site.

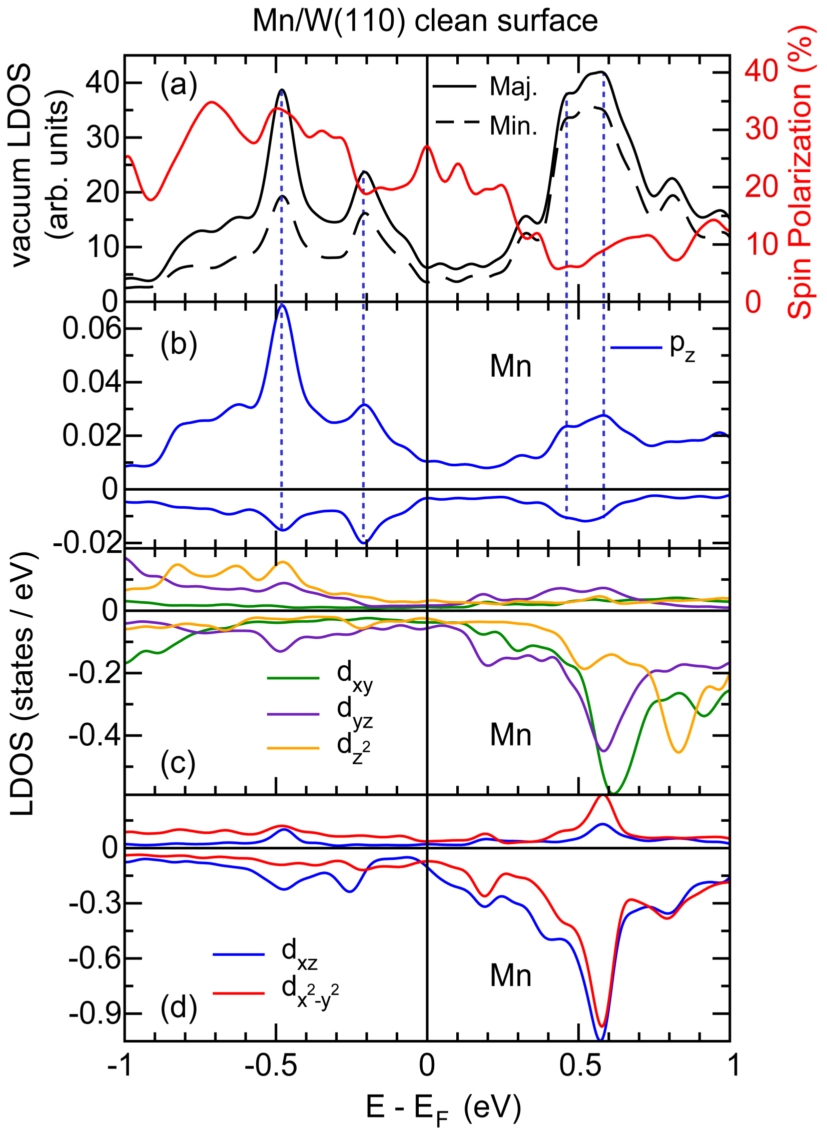

Supplementary Figure 13: Calculated LDOS above the Mn/W(110) surface. (a) Spin-resolved vacuum LDOS 5 Å above Mn/W(110) and its spin polarization. (b-d) Orbital decomposition of the LDOS of the Mn atom in terms of the majority (up) and minority (down) states.

Supplementary References

1. Serrate D., *et al.* Imaging and manipulating the spin direction of individual atoms. *Nat. Nanotechnol.* **5**, 350, (2010), doi: 10.1038/nnano.2010.64.

2. Haldar S., Heinze S. Noncollinear spin density of an adatom on a magnetic surface. *Phys. Rev. B* **98**, 220401(R) (2018), doi: 10.1103/PhysRevB.98.220401.

3. Sader J.E., Jarvis S.P. Accurate Formulas for Interaction Force and Energy in Frequency Modulation Force Spectroscopy. *Appl. Phys. Lett.* **84**, 1801-1803, (2004), doi: 10.1063/1.1667267.

4. Giessibl F.J. Atomic resolution on Si(111)-(7x7) by noncontact atomic force microscopy with a force sensor based on a quartz tuning fork. *Appl. Phys. Lett.* **76**, 1470-1472, (2000), doi: Doi 10.1063/1.126067.

5. Heinze S., *et al.* Real-Space Imaging of Two-Dimensional Antiferromagnetism on the Atomic Scale. *Science* **288**, 1805-1808, (2000), doi: 10.1126/science.288.5472.1805.

6. Heinze S., *et al.* Spontaneous atomic-scale magnetic skyrmion lattice in two dimensions. *Nat. Phys.* **7**, 713-718, (2011), doi: 10.1038/nphys2045.

7. Williams A.R., Zeller R., Moruzzi V.L., Gelatt C.D., Kubler J. Covalent magnetism: An alternative to the Stoner model. *J. Appl. Phys.* **52**, 2067-2069, (1981), doi: 10.1063/1.329617.

8. Tao K., Stepanyuk V.S., Hergert W., Rungger I., Sanvito S., Bruno P. Switching a Single Spin on Metal Surfaces by a STM Tip: Ab Initio Studies. *Phys. Rev. Lett.* **103**, 057202 (2009), doi: 10.1103/PhysRevLett.103.057202.

9. Zener C. Interaction between the d-Shells in the Transition Metals. II. Ferromagnetic Compounds of Manganese with Perovskite Structure. *Phys. Rev.* **82**, 403-405, (1951), doi: 10.1103/PhysRev.82.403.

10. Zener C. Interaction Between the d-Shells in the Transition Metals. *Phys. Rev.* **81**, 440-444, (1951), doi: 10.1103/PhysRev.81.440.

11. Zener C. Interaction between the d-Shells in the Transition Metals. III. Calculation of the Weiss Factors in Fe, Co, and Ni. *Phys. Rev.* **83**, 299-301, (1951), doi: 10.1103/PhysRev.83.299.
